# Neuro-symbolic procedural semantics for explainable visual dialogue

**DOI:** 10.1371/journal.pone.0323098

**Published:** 2025-05-27

**Authors:** Lara Verheyen, Jérôme Botoko Ekila, Jens Nevens, Paul Van Eecke, Katrien Beuls

**Affiliations:** 1 Artificial Intelligence Lab, Vrije Universiteit Brussel, Brussels, Belgium; 2 Faculté d’informatique, Université de Namur, Namur, Belgium; Shanghai Maritime University, CHINA

## Abstract

This paper introduces a novel approach to visual dialogue that is based on neuro-symbolic procedural semantics. The approach builds further on earlier work on procedural semantics for visual question answering and expands it with neuro-symbolic mechanisms that handle the challenges that are inherent to dialogue, in particular the incremental nature of the information that is conveyed. Concretely, we introduce (i) the use of a conversation memory as a data structure that explicitly and incrementally represents the information that is expressed during the subsequent turns of a dialogue, and (ii) the design of a neuro-symbolic procedural semantic representation that is grounded in both visual input and the conversation memory. We validate the methodology using the MNIST Dialog and CLEVR-Dialog benchmark challenges and achieve a question-level accuracy of 99.8% and 99.2% respectively. The methodology presented in this paper contributes to the growing body of research in artificial intelligence that tackles tasks that involve both low-level perception and high-level reasoning using a combination of neural and symbolic techniques. It thereby leads the way towards the development of conversational agents that will be able to hold more explainable, natural and coherent conversations with their human interlocutors.

## Introduction

Visual dialogue refers to the task in which an artificial agent and a human hold a meaningful and coherent conversation that is grounded in visual input [1]. Typically, an agent needs to answer a sequence of questions about a given image, where the questions can only be understood in relation to previous question-answer pairs. In many respects, the task of visual dialogue is similar to the task of visual question answering [2], with the additional difficulty that the question-answer pairs are not independent from each other.

**Fig 1:**
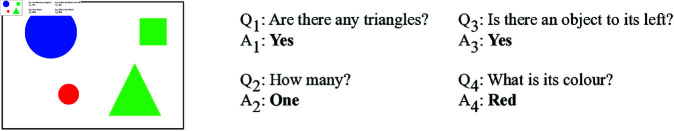
Schematic representation of a typical visual dialogue task. In this task, an artificial agent needs to answer a sequence of follow-up questions about an image.

A schematic depiction of a typical visual dialogue task is shown in Fig 1. In this example, an agent is presented with the image on the left, and needs to answer the sequence of questions ${\text{Q}}_{1}$ to ${\text{Q}}_{4}$ on the right. The four question-answer pairs constitute a coherent dialogue, in which ${\text{Q}}_{1}$ (‘*Are there any triangles?*’) can be answered based on the image alone, but in which ${\text{Q}}_{2}$ to ${\text{Q}}_{4}$ (‘*How many?*’, ‘*Is there an object to its left?*’, ‘*What is its colour?*’) can only be answered based on the combination of the image and the previous question-answer pairs.

In this paper, we introduce the use of neuro-symbolic procedural semantic representations for solving visual dialogue tasks. We build further on earlier work in the area of visual question answering, in which procedural semantic representations, as pioneered by amongst others [3,4] and [5], have already been successfully used for representing the meaning of questions in the form of executable queries [6–8]. Such procedural semantic representations capture the logical structure underlying a question, and can be executed on a given image to compute an answer.

**Fig 2:**
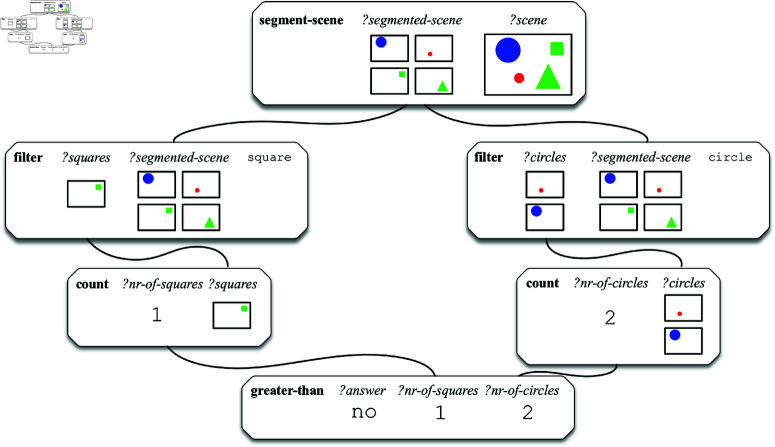
Example of a procedural semantic representation for the question ‘Are there more squares than circles?’, executed on the image in Fig 1. The answer to the question given this image is no.

An example of a procedural semantic representation for the question ‘*Are there more squares than circles?*’, asked about the image in Fig 1, is shown in Fig 2. The query is composed of six operations that need to be performed by an artificial agent in order to retrieve the answer to the question. First of all, the segment-scene operation segments the image that it received as input (bound to the variable ‘*?scene*’) and binds the set of foreground objects to the ‘*?segmented-scene*’ variable. Then, two filter operations take this set of objects as input and bind the set of squares and the set of circles to the variables ‘*?squares*’ and ‘*?circles*’ respectively. Then, the set of squares and the set of circles are counted by count operations and the cardinality of each set is computed. Finally, the greater-than operation checks whether the cardinality of the first set is larger than the cardinality of the second set. The result of this last operation (in this case no) is at the same time the answer to the question as a whole.

The operations, which are also called *primitive operations* or *primitives*, correspond to atomic actions that an artificial agent can perform. Depending on the techniques used for implementing these operations, procedural semantic representations can be *subsymbolic*, cf. the neural module networks used by [6], or *symbolic*, cf. the set operations used by [9]. In this paper, we combine the strengths of both subsymbolic and symbolic operations through the introduction of *neuro-symbolic procedural semantic representations* (cf. [10]).

When moving from visual question answering to visual dialogue, the two-step process of first mapping a question to its logical structure and then executing the corresponding query on an image becomes more challenging. As individual questions are no longer independent from each other, they no longer map onto queries that are directly executable on an image alone. For example, in the question ‘*What is its colour?*’, the possessive anaphoric pronoun “its” refers to an object that was introduced by an earlier question-answer pair, and which must be retrieved in order to be able to answer the question. As opposed to visual question answering systems, visual dialogue systems thus need to be able to keep track of the information that has been conveyed during earlier dialogue turns, as well as to use this information for answering questions in later turns.

In order to overcome this challenge, we introduce the use of a *conversation memory* as a data structure that explicitly and incrementally stores the information that is expressed in the subsequent turns of a dialogue. Additionally, we present a procedural semantic representation for visual dialogue tasks, which is able to query both visual input and the conversation memory. Due to its neuro-symbolic nature, this semantic representation can exploit the strengths of both subsymbolic systems for interacting with perceptual data, in this case the image, and of symbolic systems for reasoning based on previously acquired knowledge, in this case by retrieving structured information from the conversation memory. The evaluation of our novel methodology on the standard MNIST Dialog benchmark [11] and the more challenging CLEVR-Dialog benchmark [12] shows that through the introduction of a conversation memory and the design of a compatible neuro-symbolic procedural semantic representation, we have been able to transfer the success of using procedural semantics in the field of visual question answering to the much more challenging field of visual dialogue.

By presenting a methodology that tackles visual dialogue tasks by reasoning over both structured (memory) and unstructured (image) data, this paper contributes to the growing body of research in artificial intelligence that tackles tasks that involve both low-level perception and high-level reasoning using a combination of neural and symbolic techniques [10,13,14]. It thereby bears the promise of leading to the development of artificial agents with more explainable, consistent and human-like cognitive capacities. This paper is supplemented by an interactive web demonstration accessible at https://ehai.ai.vub.ac.be/demos/visual-dialog. A conference paper reporting on the results discussed in this paper was previously published as [15].

## Background and related work

This section sketches the background and prior work that forms the backbone of the research reported on in this paper. In particular, we focus on the state of the art in the fields of visual dialogue and procedural semantics.

### Visual dialogue

Agents holding coherent conversations with humans about the scenes they observe has been a central topic in the field of artificial intelligence since its inception in the 1950s, with SHRDLU [3] and Shakey [16] being the most notable early systems developed. More recently, also the machine learning community has become increasingly interested in the topic of artificial agents holding coherent conversations with humans about visual content. This has led to the establishment of the standardised task of *visual dialogue* as introduced by [1], and subsequently to a number of dedicated datasets and benchmark challenges, including VisDial [1], MNIST Dialog [11] and CLEVR-Dialog [12]. The task of visual dialogue can be seen as an extension of the task of visual question answering [2]. While both tasks involve answering questions about images, the questions in a visual dialogue task are organised in a coherent conversation and can involve reference to entities introduced by earlier question-answer pairs. The additional challenge faced by visual dialogue systems amounts thus to taking into account earlier dialogue turns when answering later questions.

The state of the art in visual dialogue is dominated by attention-based neural network approaches, which mainly differ in how they deal with co-references between question-answer pairs. In general, these approaches use an encoder-decoder architecture [17], which learns to attend to those regions of the image and/or previous question-answer pairs that are most relevant to answering a given question. [1] introduce encoders based on late fusion, hierarchical encoding [18] and memory networks [19]. These encoders encode the question, textual history and image, and identify those parts of the textual history that are most relevant to answering the question. A discriminative decoder can then be used to rank candidate answers, or a generative decoder can be used to produce an answer. [20] present history-conditioned image attentive encoders which do not only encode the question, textual history and entire image, but also attend over specific regions in the image that played a role in the dialogue history. [21] integrate answer options as early input to the model, as to maximally exploit their informativeness. Building further on this approach, [22] introduce a two-stage process, in which the candidate answers are first scored by a co-attention network. This ranking is then passed as input to a second co-attention network during a so-called synergistic stage. [23] propose a co-attention encoder which jointly reasons over the input image, the question and the previous question-answer pairs. This encoder is in turn part of a larger architecture for adversarial learning, which learns to approximate human answers using a reinforcement learning-based discriminator. [24] extend this work by presenting a more general co-attention-based model that can include any number of input modalities. [25] propose the use of dual attention networks for resolving visual co-references. Linguistic co-references are resolved by a first attention module, and the corresponding entities are then grounded in the image by a second attention module. [26] introduce a recurrent dual attention network that performs multi-step reasoning, integrating visual and textual reasoning in an iterative process. [27] introduce an algorithm that recursively traverses earlier question-answer pairs based on co-references, in order to retrieve visual attentions for the relevant entities. [28] propose a graph neural network approach to visual dialogue, where the nodes are dialogue turns and the edges represent co-reference links between these turns. Answering a question amounts then to inferring unknown node values. [29] present a history-aware co-attention network that is robust against imperfect history input. Their learning approach, called history-advantage sequence training, is inspired by actor-critic methods in reinforcement learning in the sense that it includes an adversarial critic which intentionally introduces wrong answers with the goal of improving robustness. [30] propose a weighted likelihood estimation method for training generative decoders, with the goal of making them less biased towards frequent answers such as ‘*I don’t know*’. [31] integrate pre-trained BERT language models into a transformer-based encoder. [32] extend this approach by integrating soft linguistic constraints, encoding preference for specific part-of-speech tags and closeness between pronouns and their antecedents.

A next line of research focuses on more explicitly keeping track of the entities that were evoked in earlier dialogue turns, both visually and textually, and on resolving co-references and ambiguities with respect to these entities. Starting from the observation that the proportion of follow-up questions with non-trivial co-references in existing visual dialogue datasets, in particular VisDial, is limited [33,34,11] introduce the MNIST Dialog dataset with the specific purpose of evaluating to what extent visual dialogue models are actually capable of reasoning about previously introduced discourse entities. MNIST Dialog is characterised by a large proportion of interdependent questions that are highly ambiguous with respect to synthetically generated scenes, unless co-references are adequately resolved. As the scenes and questions are bias-free, the questions cannot be answered without reasoning about both the scene and dialogue history. In the same paper, the authors introduce a model that explicitly represents the dialogue history as a combination of previous question-answer pairs and their associated attentions, and is able to retrieve the relevant attention for a given question from this associative memory. Building further on this work, [35] also represent the dialogue history in the form of an associative memory, but the keys are here more fine-grained entity-level descriptions instead of question-answer pairs. The authors introduce a neural module network architecture [36] in which the meaning representation includes two dedicated modules (refer and exclude) for interacting with the associative memory. [37] extend [35]’s model with a separate treatment of personal and impersonal pronouns. [12] introduce the CLEVR-Dialog dataset for studying and benchmarking multi-turn reasoning in visual dialogue. This dataset was developed as a more challenging alternative to MNIST Dialog, where questions cannot only depend on the previous question-answer pair, but also on any combination of earlier question-answer pairs. [38] introduce three extensions of memory, attention and composition (MAC) networks [39] that deal with the conversational nature of visual dialogue tasks. A first extension consists in passing information across dialogue turns by initialising the memory state of the first MAC-cell of each turn with the value of the memory state of the last MAC-cell of the previous turn. A second extension concerns a context-aware attention mechanism that implements a transformer-like self-attention mechanism on the previous control states. A final extension consists in appending the entire dialogue history to the current question. They report that all three techniques lead to important improvements with respect to the state of the art. [40] present a neuro-symbolic approach that combines deep learning and symbolic program execution for tackling visual dialogue tasks and demonstrate their methodology on the CLEVR-Dialog dataset. Their method relies on a pre-trained Mask R-CNN [41] to convert the dataset’s images to symbolic scene graphs (similar to work on the CLEVR benchmark by [42]), which they combine with a symbolic program executor and a dynamic knowledge base to keep track of the previously mentioned entities.

### Procedural semantics

Procedural semantic representations, as pioneered by [43,3] and [5], capture the meaning of linguistic expressions in the form of programs that can be executed algorithmically. The use of procedural semantics is of particular interest to conversational agents, especially when these agents need to be able to truly understand linguistic expressions as uttered by a human, for example in the case of instructions to be carried out in the world or questions to be answered in natural language. The procedural semantics paradigm was indeed the result of a number of ambitious research projects in this direction in the 1960s and 1970s. The SHRDLU system [3] was able to hold coherent conversations with a human about a blocks world. It could move blocks as instructed by the human, reason about actions and affordances, and answer questions about both actions and the state of the world. SHRUDLU’s rule-based grammar and reasoning system were not only able to understand and produce English utterances, but could also ask for clarifications when the system was unable to disambiguate input utterances. The LUNAR system [4] enabled lunar geologists to query chemical analysis data on lunar rock and soil composition using natural language, without having to learn a formal query language or the structure of NASA’s databases. English utterances were analysed by an augmented transition network (ATN)-based parser and then mapped onto queries that could be executed on the databases. [44] took this approach further, by introducing semantic transition networks (STNs). As compared to ATNs, STNs are able to directly build up a semantic representation, instead of needing to pass through an intermediate syntactic structure. Since then, this pioneering work has given rise to a broad spectrum of procedural semantics-based question answering systems. While the coverage and applicability of these systems have drastically improved over time, the conversational aspects that were once the hallmark of SHRDLU, have gradually moved away from the focus of attention.

Over the last decades, procedural semantic representations have been extensively used in systems for querying databases using natural language, in combination with a variety of grammar formalisms. [45] introduce the use of an extension of definite clause grammars [46], called extraposition grammars, to parse natural language questions into logic-based executable queries. [47] introduce an inductive logic programming approach to learn definite clause grammars and [48] uses definite clause grammars to parse natural language questions into efficient datalog queries. A large body of work embraces combinatory categorial grammar (CCG) [49] as a semantic parsing engine that maps natural language utterances onto logical forms expressed in the lambda calculus [50–56]. Other work adopts Head-Driven Phrase Structure Grammar (HPSG) [57,58], computational construction grammar [9,59], dependency parsing [36] or variations on context-free grammars [60,61]. Apart from grammar-based approaches, also neural approaches have been used to map questions onto executable queries, in particular using recurrent neural networks such as LSTMs [6,62–64].

When it comes to the properties of the procedural semantic representations themselves, three different approaches can be distinguished. A first class of models represent the meaning of utterances as queries expressed in a database querying language, such as SQL [63], FunQL [64] or SPARQL [65]. The main advantage of this approach is that the expressiveness of the semantic representation coincides with the expressiveness of the query language, and that the semantic representations can be directly executed on a database. The main disadvantages of this approach are that only questions can be represented straightforwardly and that the structure of the queries is often far removed from the way in which information is represented in natural language. A second class of models represent the meaning of questions using logical forms, often defined in terms of variations on the lambda calculus (see e.g. the work cited above in the context of CCG). Such representations are more expressive, can represent more sentence types, and more closely mirror the compositional nature of linguistic utterances. However, an additional step is needed to transform the logical forms to executable queries. The third class of models use formalisms that were especially designed to represent the meaning of natural language utterances using procedural semantic representations. These formalisms typically provide a way to define so-called *primitive operations*, which correspond to functions or predicates that can be implemented computationally. These primitive operations can be compositionally combined into larger programs, often called *semantic networks*, through shared input and output arguments. These programs can then be evaluated by executing the individual primitive operations while propagating the appropriate arguments from one operation to the other. Examples of models of this class include meaning representations expressed in Incremental Recruitment Language (IRL) [66,67], as used for example by [68] and [9], or the meaning representations used by [6,69] and [70]. While the primitive operations used in these special-purpose procedural semantics languages need to be implemented or learnt, this approach has the advantage that the languages are open-ended and directly executable. Moreover, this means that the procedural semantic languages can be tailored towards the task at hand, and that the primitive operations and their combination can be designed to better reflect the compositional nature of natural language utterances.

Primitive operations in procedural semantics can be operationalised symbolically or subsymbolically. Subsymbolic primitives perform operations over numeric representations such as scalars, vectors or tensors. They usually deal with the categorisation of sensor values observed in the world, often extracted from images. Symbolic primitives on the other hand perform operations over meaningful symbols, typically implementing higher-level reasoning processes. Neuro-symbolic procedural semantic systems allow to combine symbolic and subsymbolic primitives in semantic networks. In these networks, subsymbolic primitives typically deal with perception tasks, while symbolic primitives typically deal with reasoning tasks. Procedural semantic representations of all three types have been proposed. Neural module networks have been introduced by [36] as an operationalisation of fully subsymbolic procedural semantic representations applied to visual question answering tasks. [35] extend this approach to visual dialogue by adding primitive operations that perform multi-turn co-reference resolution. [42,71] and [9] present a symbolic approach where the procedural semantic representations are not executed on the image directly, but on a scene graph representation that is generated first. Finally, [72] and [10] propose a neuro-symbolic procedural semantic engine which integrates neural predicates in probabilistic logic programs and [14] present a framework aimed at representing fully differentiable logic representations.

## Methodology

Our novel approach to visual dialogue operationalises two main ideas. First, the history of a dialogue is represented explicitly, incrementally and in a structured way. We refer to the data structure holding this information by the term *conversation memory*. Second, the meaning of linguistic utterances is represented using a *neuro-symbolic procedural semantic representation* that combines subsymbolic and symbolic primitive operations.

### Conversation memory

The conversation memory captures all information about the dialogue history that can be relevant for interpreting later dialogue turns. It represents this information in an explicit, human-interpretable way, and is incrementally extended after each dialogue turn. Per turn, the conversation memory stores:

- a timestamp capturing the turn number.
- the utterance observed during the turn.
- the sentence type of this utterance, indicating for example the question type for questions.
- the reply that was produced, if applicable.
- the topic of the conversation from an information structure point of view.
- a symbolic representation of the set of all entities evoked during the dialogue up to this turn, including all their properties that were mentioned.
- for each entity, a pointer to an attention over the image that highlights its grounding in the input.

**Fig 3:**
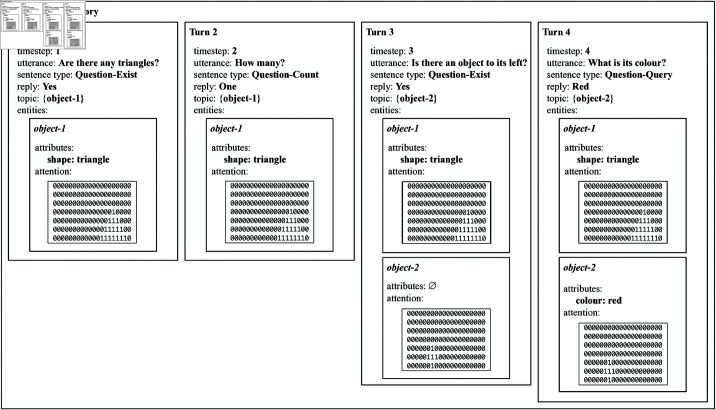
Schematic representation of the conversation memory after the fourth turn of the dialogue sketched in Fig 1. The conversation memory is incrementally updated after each dialogue turn as new information becomes available.

As an example, Fig 3 shows the state of the conversation memory after processing the dialogue introduced in Fig 1. For now, we only briefly introduce the conversation memory data structure. In the first turn, the question ‘*Are there any triangles?*’ of type Question-Exist is observed and the answer ‘*Yes*’ is returned. The topic of the conversation at this point is the entity ‘*object-1*’. Both the grounding of entity ‘*object-1*’ in the input image and its mentioned shape property are stored in the conversation memory. In the second turn, the question ‘*How many?*’ of type Question-Count is asked about the current topic of the conversation and the answer ‘*One*’ is returned. The topic of the conversation does not change and no additional information is added. In the third turn, the question ‘*Is there an object to its left?*’ of type Question-Exist is processed and the answer ‘*Yes*’ is returned. A new entity ‘*object-2*’ is added to the conversation memory with as only information its grounding in the input image. The topic of the conversation now shifts to entity ‘*object-2*’. Finally, at the fourth turn, the question ‘*What is its colour*?’ is processed. The topic of the conversation, namely ‘*object-2*’, is inferred from the previous turn and the answer ‘*Red*’ is returned. The colour property of ‘*object-2*’ is added to the representation of this entity in the conversation memory.

In general, the conversation memory should store after each dialogue turn all discourse information that might be relevant for interpreting later dialogue turns. The information that we include in our implementation of the conversation memory reflects the information that is relevant in the visual dialogue tasks that we tackle in this paper. We do not claim in any way that this information is sufficient to model everyday conversations between human interlocutors, which fall outside the scope of these benchmark challenges. Indeed, further research in pragmatics is needed in order to construct more accurate models of the role that discourse information plays in human conversation.

### Neuro-symbolic procedural semantics

In tandem with the conversation memory, we introduce a neuro-symbolic procedural semantic representation that is designed to represent the meaning of utterances in their discourse context. The set of primitive operations that is part of our semantic representation is an extension of the set of predicates used in the annotation of the CLEVR VQA dataset [69]. On the one hand, this extension was made for the procedural semantic representation to be applicable to a larger number of datasets, and on the other hand to be able to deal with the conversational aspects of dialogue through the consultation of information stored in the conversation memory.

Our neuro-symbolic procedural semantic representation combines subsymbolic primitives that implement operations over unstructured data, in particular input images or attentions, with symbolic primitives that implement operations over structured data, in particular information contained in the conversation memory. Primitives that can operate on both structured and unstructured input have both a symbolic and a subsymbolic implementation. At runtime, the adequate implementation is then chosen based on the type of the input arguments.

**Fig 4:**
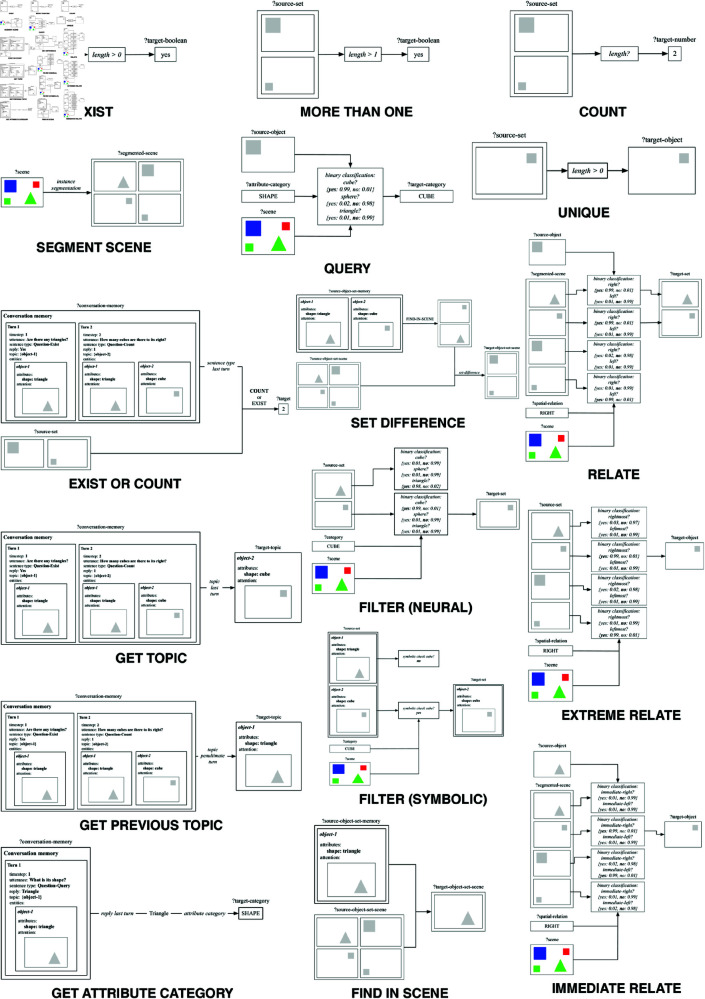
Schematic representation of the implementation of the primitive operations.

The neuro-symbolic procedural semantic representation makes use of 16 primitive operations, which can combine to represent the meaning of statements and questions about objects in an image. The statements and questions can be about the existence and number of objects in the image, their attributes and the spatial relationships between the objects. The primitive operations are defined and implemented as described below. A schematic representation of the internal architecture of each primitive operation is also provided in Fig 4 and an overview of the different primitive operations as categorised by their symbolic or subsymbolic nature is shown in Table 1.

**Table 1:** Overview of primitive operations categorised by their symbolic or subsymbolic implementation.

Symbolic	Subsymbolic
filter	filter
unique	segment-scene
count	relate
exist	extreme-relate
more-than-one	immediate-relate
exist-or-count	query
get-topic	
get-previous-topic	
get-attribute-category	
find-in-scene	
set-difference

- The segment-scene(?segmented-scene, ?scene) primitive operation binds a segmentation of the input image bound to ‘*?scene*’ to the ‘*?segmented-scene*’ variable, i.e. a set of attentions in which each attention highlights one of the objects in the image. This primitive operation is implemented subsymbolically as a Mask R-CNN-based neural network that performs instance segmentation [41]. The segment-scene primitive is used in the representation of the meaning of each statement or question about an image. For example, it serves as a starting point for computing an answer to the question ‘*Are there any green cylinders?*’.
- The filter(?target-set, ?source-set, ?scene, ?category) primitive operation binds ‘*?target-set*’ to the set of all instances of ‘*?category*’ present in ‘*?source-set*’. ‘*?category*’ needs to be bound to a conceptual category to filter by, such as ‘green’, ‘cube’ or ‘large’. The filter operation is implemented both symbolically and subsymbolically. The symbolic implementation is used to filter entities from the conversation memory by binding the set of all entities from the ‘*?source-set*’ set that have ‘*?category*’ among their symbolic attributes to ‘*?target-set*’. The subsymbolic implementation classifies each attention in ‘*?source-set*’ according to whether it fits best the class ‘*?category*’ in ‘*?scene*’ or a different class of the same attribute category. The set of attentions that are predicted to belong to class ‘*?category*’ are bound to ‘*?target-set*’. This classification process is implemented on top of the shared inventory of neural modules discussed later in this section. The subsymbolic implementation of the filter primitive is for example used to compute the set of green objects when processing the utterance ‘*Are there any green objects?*’. The symbolic implementation is for example used to compute the set of green objects when processing the utterance ‘*How many cubes are there among the aforementioned green objects?*’.
- The relate(?target-set, ?source-object, ?segmented-scene, ?scene, ?spatial-relation) primitive operation binds ‘*?target-set*’ to the set of all attentions in ‘*?segmented-scene*’ for which ‘*?spatial-relation*’ holds with respect to ‘*?source-object*’. For example, if ‘*?spatial-relation*’ is bound to ‘right’, ‘*?target-set*’ will be bound to the set of all attentions over objects that are located to the right of ‘*?source-object*’. This primitive operation is implemented on top of the shared inventory of neural modules discussed later in this section. It classifies each attention in ‘*?segmented-scene*’ according to whether it is ‘*?spatial-relation*’ with respect to ‘*?source-object*’ in ‘*?scene*’. The primitive is used for example to compute the set of objects located to the right of a green sphere when processing the utterance ‘*How many objects are to the right of the green sphere?*’.

- The extreme-relate(?target-object, ?source-set, ?scene, ?spatial-direction) primitive operation binds ‘*?target-object*’ to the attention in ‘*?source-set*’ over the object that is located most towards the spatial direction described by ‘*?spatial-direction*’. For example, if ‘*?spatial-direction*’ is bound to ‘right’, ‘*?target-object*’ will be bound to the attention over the rightmost object present in ‘*?source-set*’. This primitive operation is implemented on top of the shared inventory of neural modules discussed later in this section. The primitive is used for example to compute the rightmost object when processing the utterance ‘*What is the colour of the rightmost object?*’.
- The immediate-relate(?target-object, ?source-object, ?segmented-scene, ?scene, ?spatial-relation) primitive operation binds ‘*?target-object*’ to the attention in ‘*?segmented-scene*’ over the object in ‘*?scene*’ that is located most closely to ‘*?source-object*’ according to ‘*?spatial-relation*’. For example, if ‘*?spatial-relation*’ is bound to ‘right’, ‘*?target-object*’ will be bound to the attention over the object in ‘*?scene*’ that is located most closely to the right of ‘*?source-object*’. This primitive operation is implemented on top of the shared inventory of neural modules discussed later in this section. The primitive is used for example to compute the object that is located most closely to the right of the green sphere in the utterance ‘*What is the shape of the object right of the green sphere?*’.
- The unique(?target-object, ?source-set) primitive operation checks whether the set bound to ‘*?source-set*’ contains only one attention. If this is the case, it binds ‘*?target-object*’ to this attention. If ‘*?source-set*’ is empty, the primitive signals failure. If ‘*?source-set*’ contains more than one attention, it triggers a search process with as many branches as there are attentions in ‘*?source-set*’. Each attention in ‘*?source-set*’ is bound to ‘*?target-object*’ in exactly one branch with the average confidence score of the attention accumulated over any previous primitives taken as the heuristic value of the branch. The unique primitive is implemented through symbolic set operations. It is for example used for processing utterances that contain articles, such as ‘*What is the material of the green sphere?*’ or ‘*There is a green object left of a red object.*’.
- The query(?target-category, ?source-object, ?scene, ?attribute-category) primitive operation queries the ‘*?attribute-category*’ of ‘*?source-object*’ and binds the resulting value to ‘*?target-category*’. ‘*?attribute-category*’ needs to be bound to the name of an attribute category, such as ‘shape’, ‘colour’ or ‘size’. The resulting values are conceptual categories such as ‘block’, ‘red’ or ‘large’. This primitive operation is implemented on top of the shared inventory of neural modules discussed later in this section. Based on ‘*?attribute-category*’ (e.g. size), a subset of binary classifiers associated to this ‘*?attribute-category*’ is selected (e.g. large, small). The category associated to the binary classifier yielding the highest confidence score (for a positive result) is bound to ‘*?target-category*’. The query primitive is used to query properties of objects, for example the material of the green sphere in the utterance ‘*What is the material of the green sphere?*’.
- The count(?target-number, ?source-set) primitive operation binds the cardinality of ‘*?source-set*’ to ‘*?target-number*’. This primitive operation is implemented through a symbolic set operation. An example utterance that requires the count primitive is the question ‘*How many spheres are there?*’.
- The exist(?target-boolean, ?source-set) primitive operation checks whether the set bound to ‘*?source-set*’ contains at least one element. If so, ‘*?target-boolean*’ is bound to ‘yes’, otherwise to ‘no’. This primitive operation is implemented through symbolic set operations. An example of an utterance requiring the exist primitive is the question ‘*Are there any spheres?*’.
- The more-than-one(?target-boolean, ?source-set) primitive operation checks whether the set bound to ‘*?source-set*’ contains multiple elements (i.e. at least two). If so, ‘*?target-boolean*’ is bound to ‘yes’, otherwise to ‘no’. This primitive operation is implemented through symbolic set operations. An example of an utterance that requires the more-than-one primitive is the statement ‘*There are multiple spheres in the image.*’.
- The exist-or-count(?target, ?source-set, ?conversation-memory) primitive operation calls either the exist primitive operation or the count primitive operation on ‘*?source-set*’ and binds the result to ‘*?target*’. Whether the exist or count operation is called, depends on the sentence type of the previous turn in ‘*?conversation-memory*’. This primitive operation is implemented through symbolic operations on the conversation memory and through calls to other primitive operations. For example, if a question ‘*and to its right*?’ follows a count-type question such as ‘*How many objects are there to the left of the green cube?*’, the count primitive will be used to count the objects to the right of the green cube. If the same question follows an exist-type question such as ‘*Are there any objects to the left of the green cube?*’, the exist primitive will be called to determine whether there are any objects to the right of that green cube.
- The get-topic(?target-topic, ?conversation-memory) primitive operation binds ‘*?target-topic*’ to the current topic of the conversation as stored in ‘*?conversation-memory*’, i.e. the set of objects that is the topic of the conversation after processing the previous turn. This primitive operation is implemented symbolically. It is used to resolve anaphora in questions such as ‘*and its colour*?’, following questions such as ‘*What is the shape of the small object left of the green cube?*’, which shifted the topic to the small object left of the green cube.
- The get-previous-topic(?target-topic, ?conversation-memory) primitive operation binds ‘*?target-topic*’ to the previous topic of the conversation, i.e. the set of objects that was the topic before processing the last turn. This primitive operation is implemented symbolically. It is used to resolve anaphora in questions such as ‘*and to its left?*’ following questions such as ‘*Are there any objects to its right?*’, which follow themselves questions such as ‘*Is there a green cube?*’. In this case, the question ‘*and to its left?*’ refers to the green cube and not to the objects to the right of the green cube.
- The get-attribute-category(?target-category, ?conversation-memory) primitive operation binds ‘*?target-category*’ to the attribute category that was queried most recently in the conversation. This primitive operation is implemented symbolically and is used to resolve anaphora in utterances such as ‘*and that of the green sphere?*’ following utterances such as ‘*What is the material of the grey cylinder?*’.
- The find-in-scene(?target-object-set-scene, ?source-object-set-scene, ?source-object-set-memory) primitive operation relates one or more objects from the conversation memory with their counterparts in the input image. Concretely, this operation takes as input a set of entities stored in the conversation memory, bound to ‘*?source-object-set-memory*’, and the attentions bound to ‘*?source-object-set-scene*’. It then finds the attentions of the entities from the conversation memory in the scene and binds this set to ‘*?target-object-set-scene*’. This primitive is implemented symbolically as a straightforward lookup function. The find-in-scene primitive is used to resolve anaphora in utterances such as ‘*What is its material?*’ following utterances such as ‘*Is there a green cube?*’. Here, the find-in-scene primitive relates the representation of the green cube as retrieved from the conversation memory with the green cube as observed in the image.
- The set-difference(?target-object-set-scene, ?source-object-set-scene, ?source-object-set-memory) primitive operation binds ‘*?target-object-set-scene*’ to the subset of ‘*?source-object-set-scene*’ that contains all attentions over objects that are not part of ‘*?source-object-set-memory*’. It does this by first using the find-in-scene primitive to retrieve the attentions over the objects in ‘*?source-object-set-memory*’ and then subtracting these from ‘*?source-object-set-scene*’. This primitive is implemented through symbolic functions. The set-difference primitive is used to process utterances that explicitly refer to objects that were not previously mentioned, for example in the utterance ‘*Are there other objects sharing its colour?*’. Here, the word ‘other’ refers to the set of objects in the scene that do not appear in the conversation memory.

The subsymbolic primitive operations that query attributes of objects (query), that filter objects based on their attributes (filter), and that spatially relate objects to each other (relate, extreme-relate and immediate-relate) are all implemented on top of a shared inventory of neural modules. These modules are implemented as binary classifiers that are trained to predict whether a specific conceptual categorisation holds for a given object or set of objects in a scene. They should be interpreted as atomic distinctions that underlie the conceptual reasoning of an agent operationalised through a variety of primitive operations. Using a shared inventory of highly-specialised neural modules across different primitive operations, as opposed to training a dedicated neural module for each subsymbolic primitive operation, has two main advantages. First, it enhances the consistency of the overall reasoning process, as the different reasoning steps make use of the same conceptual representations and inferences. Second, it facilitates the addition of new primitive operations as they can maximally reuse cognitive capacities that have previously been acquired. All binary classifiers are convolutional neural networks that adopt the SqueezeNet architecture [73]. An overview of the neural modules is shown in Table 2 and full details on their implementation and evaluation are provided in the appendix of this paper.

**Table 2:** Overview of the shared inventory of neural modules on top of which the subsymbolic primitive operations are built. All modules are implemented as binary classifiers adopting the SqueezeNet architecture [73].

colour-blue?	relate-behind?	extreme-relate-right?	style-stroke?
colour-red?	relate-left?	extreme-relate-front?	style-flat?
colour-brown?	relate-right?	extreme-relate-middle?	number-0?
colour-green?	relate-front?	size-small?	number-1?
colour-cyan?	immediate-relate-behind?	size-large?	number-2?
colour-gray?	immediate-relate-left?	bgcolour-white?	number-3?
colour-purple?	immediate-relate-right?	bgcolour-cyan?	number-4?
colour-yellow?	immediate-relate-front?	bgcolour-salmon?	number-5?
colour-violet?	immediate-relate-above?	bgcolour-silver?	number-6?
shape-cube?	immediate-relate-below?	bgcolour-yellow?	number-7?
shape-cylinder?	extreme-relate-behind?	material-metal?	number-8?
shape-sphere?	extreme-relate-left?	material-rubber?	number-9?

### Extending the conversation memory

The conversation memory is extended with new information after each dialogue turn. Concretely, after each turn, a new turn representation is created for the current timestep (see the four boxes in Fig 3). The timestep, utterance and reply slots of the turn representation are straightforwardly filled based on the available information. The sentence type is inferred from the final primitive operation executed during the evaluation of the semantic network for the current utterance. The topic corresponds to the set of objects that was bound to the input argument of the same primitive operation call. Finally, entities are added or updated based on the properties of the objects that were mentioned during the current turn.

For example, in the first turn of Fig 3, the question ‘*Are there any triangles?*’ is asked and the response is ‘*Yes*’. It can be inferred that the question is of type Question-Exist based on the fact that the semantic network representing the meaning of the question ends with the exist primitive (the semantic network is not shown in the figure). The topic corresponds to the set containing the only triangle that was present in the input image, and which served as the input set to be processed by the exist primitive. A representation of this object is added to the list of entities with its mentioned ‘*triangle*’ property and an attention that grounds the object in the image. In the third turn, the question ‘*Is there an object to its left?*’ of type Question-Exist is asked and the answer ‘*Yes*’ is returned. The topic now shifts to the set containing the only object that was to the left of the previous topic, as this was the input to the exist primitive. No information apart from its grounding in the world is added to the entity representation, as no additional information was mentioned. In the final turn, the question ‘*What is its colour?*’ of type Question-Query is asked and the answer *Red* is given. The property ‘*colour: red*’ is added to the representation of the topic entity. The topic does not shift, as it was again the same object that was the input to the final Query primitive.

## Experiments

We now validate our novel methodology using two standard benchmark challenges in the field of visual dialogue, in particular MNIST Dialog [11] and CLEVR-Dialog [12]. Both benchmarks were explicitly designed to be bias-free and to include a large proportion of non-trivial co-references across dialogue turns. Due to these two characteristics, answering the questions in the datasets cannot be done based on any statistical properties of the scenes, questions and answers, but requires actual reasoning about both the visual content and the discourse context.

### MNIST dialog

#### Data.

**Fig 5:**
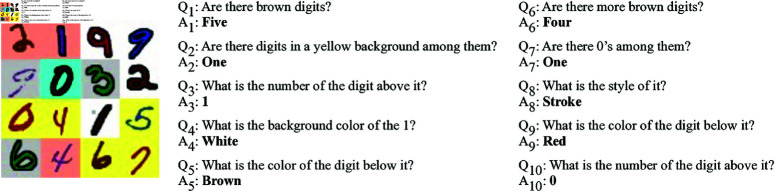
Example dialogue from the MNIST Dialog dataset.

The MNIST Dialog dataset consists of 50,000 images, which are each accompanied by three dialogues. Each dialogue is in turn composed of 10 question-answer pairs. Each image consists in a synthetically generated 4x4 grid of hand-drawn digits with four randomly sampled attributes: colour (‘*red*’, ‘*green*’, ‘*blue*’, ‘*purple*’ or ‘*brown*’), background colour (‘*cyan*’, ‘*yellow*’, ‘*white*’, ‘*silver*’ or ‘*salmon*’), number (from 0 to 9) and style (‘*flat*’ or ‘*stroke*’). A symbolic description of the scene is also provided as meta-data, but is not part of the actual benchmark. The questions and answers are automatically generated. The questions can either query attributes of a single digit (e.g. ‘*What is the colour of the digit below it?*’) or count digits based on one or more of their attributes (‘*Are there brown digits?*’^1^ ). They can also include references to the spatial relations between the digits. The answers always take the form of a single word. An example dialogue from the MNIST Dialog dataset is shown in Fig 5. [11] estimate that 94% of the questions involve co-references, either in the form of pronouns or in the form of definite noun phrases.

#### Operationalisation and experimental set-up.

There are three main challenges involved in the operationalisation of our methodology for the MNIST Dialog benchmark. First of all, we need a means to map the MNIST questions to semantic networks that are composed of the primitive operations that we have introduced above. This is a highly non-trivial task, as the MNIST dataset does not come with any semantic annotation of the questions. Second, we need to train the neural modules that are used by these primitive operations on the MNIST dataset images. Finally, we would like to be able to evaluate the process of mapping from questions to semantic networks, the execution of these networks, and the neural modules themselves independently from each other.

In order to operationalise the process of mapping from the MNIST questions to their semantic representations, we adopted a computational construction grammar-based approach [74–78]. Concretely, we extended the computational construction grammar developed by [9] for the CLEVR VQA dataset [69] so that it is able to handle constructions involving co-referential expressions. The meaning predicates contributed by these additional constructions are expressed in terms of the primitive operations defined above. Other approaches for mapping from natural language utterances to semantic networks, such as LSTM-based techniques, have also been proposed in the literature (see above), but require a gold standard annotation of the semantic networks in the dataset. Although interesting in its own right, the details of the grammar itself fall outside the scope of this paper. We refer readers interested in the application of construction grammar-based approaches to visual question answering tasks to [9]. The execution of the semantic networks is modelled using the Incremental Recruitment Language (IRL) framework [66,67,79,80], a procedural semantics implementation.

In order to verify the aptness of the semantic representations resulting from the language processing process, we have in a first phase made symbolic implementations of the primitive operations that work on the noise-free meta-data that describe the images rather than on the images themselves. By doing this, we could verify whether the predicted semantic networks would in theory always lead to the correct answer given a question and a scene. We could show that the semantic networks indeed achieved a 100% accuracy when applied to the meta-data of the images. This proves on the one hand that the primitive operations presented above are indeed sufficient to represent the meaning of the questions in the dataset, and on the other hand that our grammar covers the dataset completely. It is obviously the temporary noise-free condition of the synthetic dataset that makes the 100% figure possible.

The neural modules underlying the primitives described above were then trained on the training section of the MNIST dataset and their accuracy was evaluated on the validation set. All individual primitive operations achieved an accuracy of over 99.80% on the image data. The details of the training process and the evaluation results of the individual neural primitives are described in the appendix of this paper.

**Fig 6:**
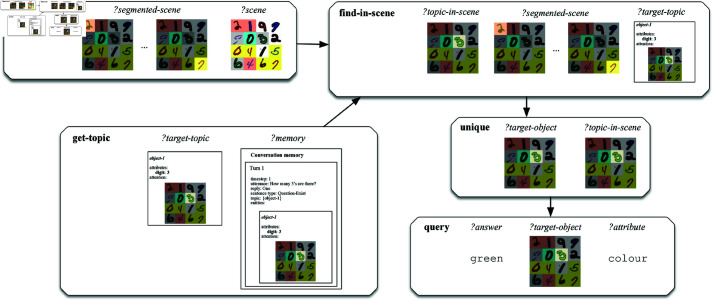
Schematic representation of the execution of the semantic representation for the utterance ‘What is its colour?’ following the utterance ‘How many 3’s are there?’ on a scene from the MNIST Dialog dataset.

An operational example of our methodology as applied to a question and scene from the MNIST Dialog dataset is shown in Fig 6. The figure shows the execution of the semantic network corresponding to the question ‘*What is its colour?*’. This question is asked as the second turn in a dialogue, following the question-answer pair ‘*How many 3’s are there? One.*’. The semantic representation is composed of five primitive operations: segmenting the image (segment-scene), retrieving the topic of the conversation from the conversation memory (get-topic), retrieving the topic in the scene (find-in-scene), checking whether the retrieved topic corresponds to a single object (unique) and querying the colour of this object (query). When it comes to the execution of this network, the get-topic primitive extracts the topic from the last turn of the conversation memory and binds the retrieved topic to the variable ‘*?target-topic*’. The segment-scene primitive binds a segmentation of the entire scene to the ‘*?segmented-scene*’ variable. The find-in-scene primitive uses the bindings of ‘*?target-topic*’ and ‘*?segmented-scene*’ to compute the topic of the previous turn in the current scene. The resulting attention, in this case highlighting a single cell in the second row on the third column, is bound to the variable ‘*?topic-in-scene*’. The unique primitive operation checks whether there is indeed a single attention in the set bound to ‘*?topic-in-scene*’ and binds the attention to the variable ‘*?target-object*’. Finally, the query primitive queries the colour attribute of the target object and binds the answer ‘*green’* to the ‘*?answer*’ variable. In terms of the classification of primitives introduced above, the segment-scene and query operations have a subsymbolic implementation, whereas the unique, get-topic and find-in-scene operations have a symbolic implementation. It is the find-in-scene operation that bridges between the symbolic and subsymbolic domains.

When it comes to evaluating the performance of the overall system on the test portion of the MNIST Dialog benchmark dataset, we include two different experimental set-ups. First of all, in the ‘*standard*’ setting, we evaluate the accuracy of the answers provided by the execution of the semantic networks that result from language processing. In the ‘*guessing*’ setting, the system is allowed to make an educated guess when the execution of a semantic network fails and therefore does not lead to an answer. The educated guess is made based on the question type as identified by the grammar and the distribution of answers per question type in the training set. For example, if the question ‘*What is the colour of the 6?*’ is asked and the conversation memory does not contain a reference to any 6s, for example due to a previous classification error, the execution of the semantic network fails and a guess is made based on the distribution of colours as answers in the training data. The ‘*guessing*’ setting is provided in order to be able to straightforwardly compare our results to neural approaches which always provide an answer even if its probability is low. The experimental results obtained on the MNIST Dialog dataset are provided in Table 3 and will be discussed in the discussion section.

**Table 3:** Overview of results for MNIST Dialog, CLEVR-Dialog and CLEVR VQA.

	MNIST Dialog	CLEVR-Dialog	CLEVR VQA
*Encoder-decoder approaches*			
emsp; LF [1]	45.1	55.9	/
emsp; HRE [1]	49.1	63.3	/
emsp; MN [1]	48.5	59.6	/
emsp; AMEM [11]	96.4	/	/
*Neural module networks*			
NMN^2^ [36]	/	/	72.1
emsp; IEP [7]	/	/	96.9
emsp; N2NMN^3^ [81]	23.8	56.6	83.7
emsp; TbD [82]	/	/	99.0
emsp; corefNMN [12]	99.3	68.0	/
*MAC networks*			
emsp; MAC [39]	/	/	98.9
emsp; MAC-CQ-CAA-MTM [38]	/	98.3	/
*Neuro-symbolic approaches*			
emsp; NS-VQA [42]	/	/	**99.8**
emsp; NS-Visdial [40]	/	**99.7**	/
*Ours*			
emsp; standard	**99.8**	99.0	99.7
emsp; guessing	**99.8**	99.2	99.7

### CLEVR-Dialog

#### Data.

**Fig 7:**
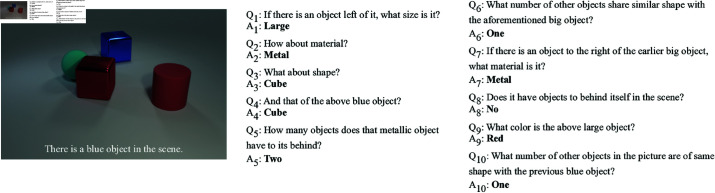
Example dialogue from the CLEVR-Dialog dataset.

The CLEVR-Dialog dataset consists of 85,000 images, which are each accompanied by five dialogues. Each dialogue starts with a caption that makes a statement about the contents of the image (e.g. ‘*There is a gray object right of a large object*’). The caption is then followed by 10 question-answer pairs. The images depict synthetically generated scenes consisting of 3D geometrical objects with randomly sampled attributes: shape (‘*cube*’, ‘*sphere*’ or ‘*cylinder*’), size (‘*small*’ or ‘*large*’), colour (‘*green*’, ‘*red*’, ‘*gray*’, ‘*blue*’, ‘*brown*’, ‘*yellow*’, ‘*purple*’ or ‘*cyan*’) and material (‘*rubber*’ or ‘*metal*’). The questions involve querying an attribute of an object in the scene (e.g. *‘What shape is it?’*), counting objects based on one or more of their attributes (e.g. ‘*How many green spheres are there?*’), and querying whether a set of objects satisfies a given description (e.g. ‘*Are there any green spheres?*’). The questions can involve reference to different kinds of spatial relations between objects (e.g. ‘*the left block*’ and ‘*the block left of the green cylinder*’). In contrast to MNIST Dialog questions, anaphora in CLEVR-Dialog questions can refer to entities mentioned in any of the previous dialogue turns. Moreover, resolving history-dependent questions can require taking into account the entire dialogue history, as is for example the case in questions such as ‘*How many other objects are present in the image?*’. An example dialogue from the CLEVR-Dialog dataset is shown in Fig 7.

#### Operationalisation and experimental set-up.

The challenges involved in operationalising our methodology for the CLEVR-Dialog benchmark are the same as those discussed above in the context of the MNIST Dialog benchmark: (i) mapping the CLEVR-Dialog questions to semantic networks that are composed of the primitive operations introduced above, (ii) training the neural modules underlying these operations on the CLEVR-Dialog images, and (iii) evaluating the accuracy of the language processing system and the neural modules.

In order to map from utterances to procedural semantic networks, we use the exact same construction grammar as the one used for the MNIST Dialog benchmark. In order to verify the aptness of the programs and language processing system, we create temporary symbolic implementations of the primitives and evaluate the programs that resulted from language processing on the noise-free meta-data that describe the images in the dataset. We achieved an accuracy of 99.99%. After an exhaustive error analysis, we could conclude that the non-perfect accuracy was due to scenes that contained an even number of objects and in which a question relied on reference to the object ‘in the middle’^4^. As the dataset was constructed in such a way that these questions are impossible to answer reliably, even for a human, we concluded that the primitives are sufficient to solve the task of CLEVR-Dialog, and that the grammar achieves maximum coverage on the CLEVR-Dialog questions.

The neural modules underlying the primitive operations described above were trained on the training portion of the CLEVR-Dialog dataset and their accuracy was evaluated on a held-out validation set of 10,000 images. All modules except the ‘extreme-relate-middle?’ module achieved an accuracy of over 99.7%. The lower accuracy of this module (97.59%) is probably due to the previously described problem in which a question can refer to the ‘middle’ object in a scene with an even number of objects. The details of the training process and the evaluation results of each individual module are described in the appendix of this paper.

**Fig 8:**
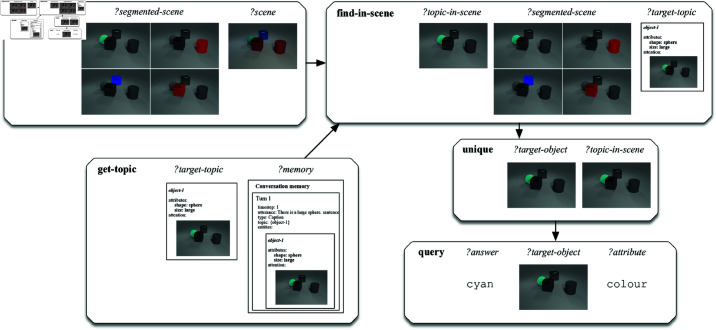
Schematic representation of the execution of the semantic representation for the utterance ‘What is its colour?’ following the caption ‘There is a large sphere.’ on a scene from the CLEVR-Dialog dataset.

An operational example of the execution of a semantic network underlying a question from the CLEVR-Dialog dataset on an image is shown in Fig 8. In this example, the same question as in Fig 6 is asked, namely ‘*What is its colour?*’. However, in this case the question follows the caption ‘*There is a large sphere.*’. Also, the question is now asked about a 3D rendered image rather than about a 2D 4x4 grid. The grammar maps the question to the same underlying procedural semantic program consisting of five primitive operations. However, the implementations of these primitives now make use of the neural modules trained on the CLEVR-Dialog images. The primitive operations are executed and the answer ‘*cyan*’ is returned.

In order to evaluate the performance of the overall system on the test portion of the CLEVR-Dialog benchmark dataset, we make use of the two same experimental set-ups as for the MNIST Dialog dataset. In particular, we provide the ‘*standard*’ and ‘*guessing*’ settings. The experimental results obtained on the CLEVR-Dialog dataset are provided in Table 3 and will be discussed here below.

## Results and discussion

An overview of the evaluation results of our system on the MNIST Dialog and CLEVR-Dialog benchmark datasets is shown at the bottom of Table 3. In the best-performing experimental setting, i.e. the ‘*guessing*’ setting, where the system makes an educated guess when the execution of a semantic network fails, our system achieves a question-level accuracy of 99.8% on the MNIST Dialog benchmark and of 99.2% on the more challenging CLEVR-Dialog benchmark. In the ‘*standard*’ setting, i.e. without guessing, it achieves a question-level accuracy of 99.8% and 99.0% respectively. The table also reports on the system’s performance on the standard CLEVR VQA benchmark, with a question-level accuracy of 99.7%. CLEVR VQA is not a visual dialogue benchmark, but has been included for reference as it has been a very popular benchmark in the literature.

The table also compares our results against previous approaches, namely the encoder-decoder-based approaches presented by [1] and [11], the neural module networks-based approaches by [36,7,81,82] and [12], the MAC network-based approaches by [39] and [38], and the neuro-symbolic scene-graph-based approaches by [42] and [40]. We can see that our system outperforms the state-of-art on MNIST Dialog and obtains near-state-of-the-art performance on CLEVR-Dialog and CLEVR VQA. While other approaches that tackle both visual dialogue benchmark challenges typically perform much better on the easier MNIST Dialog benchmark as compared to more challenging CLEVR-Dialog benchmark, our approach obtains consistently good results across both visual dialogue datasets.

While the reported benchmark accuracies are definitely important to validate our methodology in comparison to existing approaches, the more prominent contribution of the methodology that we present lies in four main characteristics that distinguish it from the state of the art in visual dialogue. First of all, the methodology is explainable in human-interpretable terms. Input utterances are mapped onto procedural semantic representations, which correspond to logic programs. These programs, which reveal the logical structure underlying an input utterance, are composed of human-interpretable primitive operations, such as count, query and filter. This means that the result of the initial language processing step can be inspected and understood by the user. The conversation memory of the system also stores information about the history of a dialogue in a structured and human-interpretable way, thereby being fully transparent about what is remembered by the system. The input and output of each primitive operation can be traced and interpreted, as they consist in either meaningful symbols (human-interpretable categories) or visual attentions over images. Given that these visual attentions are the input and output of human-interpretable operations, humans are able to judge whether an attention corresponds to what is expected or not. As the symbolically implemented primitives can be traced on a meaningful level, the only aspect of the system where the interpretability of the computation is limited is situated in the subsymbolic primitives that deal with perception on the lowest level. By pushing the neuro-symbolic boundary so far down, we ensure that any reasoning capabilities that exceed the perception of basic categories is explainable in human-interpretable terms.

A related advantage of this approach is that it avoids inconsistencies in reasoning by implementing its subsymbolic primitive operations on top of a shared inventory of highly-specialised neural modules. Keeping consistency across reasoning operations is a highly desirable property of intelligent systems, which at the same time leads to a more human-like behaviour. For example, it is obvious that the human capabilities of recognising objects and counting objects rely on the same conceptual distinctions. This is reflected in our system by implementing the count primitive in terms of computing the cardinality of a set of objects returned by a filter operation, which is itself implemented based on the same set of binary classifiers as the query operation. The answer to the question ‘*How many red blocks are there?*’ is as a consequence guaranteed to be consistent with the answers to the question ‘*What is the colour of the block?*’ asked for each block in the scene.

A third asset of our approach is that it can effectively monitor its own performance. This has become a topic of high interest in the AI community, since deep neural networks often provide confidence scores of poor quality, especially when it comes to out-of-distribution data [83,84]. Concretely, in our case, the system knows that it has not been able to answer a question based on sound logic reasoning if the execution of a semantic network fails. While it can still make an educated guess in such cases, the system then indicates that the result should be interpreted with extra care. In fact, the execution of a semantic network fails in 55.0% of the CLEVR-Dialog errors (i.e. errors in the ‘standard’ setting) and in 41.7% of the MNIST Dialog errors (in the ‘standard’ setting as well). The remaining 45.0% and 58.3% of errors respectively remain undetected by the system. This amounts to only 0.4% of the questions in CLEVR-Dialog and 0.1% of the questions in MNIST Dialog.

A final advantage resides in the modularity of the approach. New primitive operations can be added to the system in order to accommodate new tasks or to model new cognitive capabilities acquired by an artificial agent. These new primitives can add to both the logical and perceptive reasoning capabilities of the agent. Where appropriate, they can reuse neural modules used by existing primitives without needing to retrain them. Neural modules can also dynamically be added, but these might affect the performance of other modules and therefore require retraining some of them. For example, adding a binary classifier for a new colour will likely affect the performance of existing binary classifiers for other colours, as these were trained in the absence of the new colour category.

When applying the methodology to a new domain or dataset, two aspects remain particularly challenging. The first one concerns the design of an adequate inventory of primitive operations. In general, such an inventory can be modelled after what is known about human cognition, can be based on a particular theory of computation, or can more practically result from system constraints such as a robot’s API [85]. The second challenge concerns learning to map from questions to procedural semantic representations that combine primitive operations. For a deeper discussion of this topic, we refer the interested reader to [8,86].

**Fig 9:**
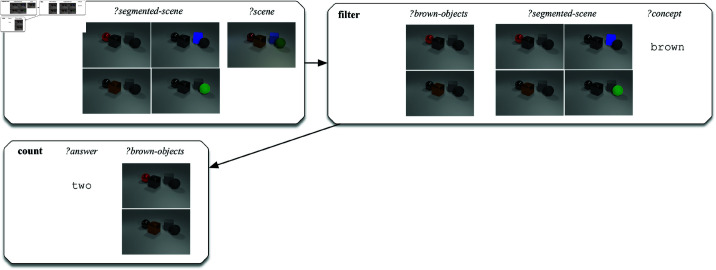
Schematic representation of the execution of the semantic network underlying the utterance ‘How many brown objects are there?’ on a scene from the CLEVR-Dialog dataset, illustrating the transparency of the approach. The filter operation wrongly recognises the leftmost object to be brown. As a consequence, two brown objects are counted instead of one.

**Fig 10:**
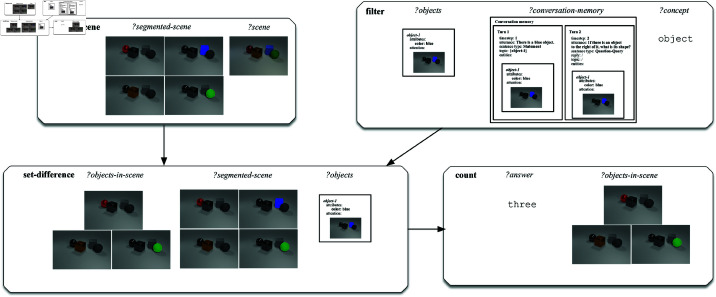
Schematic representation of the execution of the semantic network underlying the utterance ‘How many other objects are there?’ on a scene from the CLEVR-Dialog dataset, illustrating the transparency of the approach. The figure shows that the semantic network and its execution are flawless. As a consequence, the erroneous answer three must be due to an error in the conversation memory introduced in a previous dialogue turn.

Figs 9 and 10 illustrate the interpretability of our approach by providing two examples of questions from the CLEVR-Dialog dataset that were wrongly answered. Concretely, these examples show how the system supports the tracking of the source of errors by providing insight into the logical structure underlying the question, and into the input and output of the different primitive operations that were performed. Fig 9 shows the execution of the semantic network underlying the utterance ‘*How many brown objects are there?*’ on a given CLEVR scene. We can see that the question has been analysed into three primitive operations: segmenting the scene (segment-scene), filtering the segmented scene for the colour brown (filter) and counting the number of the resulting set of brown objects (count). The result of the counting operation, which is at the same time returned as the answer to the question, is two. However, this answer does not match the gold standard answer from the dataset, which is one. Indeed, when scrutinising the execution trace of the semantic network on the scene, it becomes clear that the filter operation has retrieved two brown objects. After a visual inspection of the attentions, the human observer can see that the leftmost object in the scene was wrongly classified as being brown and the source of the error has been found. If we would now query the colour of the leftmost object in the scene, the system is also guaranteed to answer brown, as the filter and query primitives internally rely on the same neural classifiers. Thus, while the answer to the question is wrong, it is logically consistent with the overall perception and reasoning skills of the system.

Fig 10 traces back the source of the erroneous answer three to the question ‘*How many other objects are there?*’. We can see that the question is analysed into four primitive operations: segmenting the scene (segment-scene), filtering the conversation memory for objects (filter), taking the set difference between the objects in the segmented scene and those retrieved from the conversation memory, and counting the resulting set (count). In this case, the conversation memory spans two turns in which only a single object has been mentioned. Indeed, the scene contains three objects apart from the one that has been mentioned already. All aspects of the construction and execution of the semantic network seem to be flawless, but the answer three does not match the gold standard answer two. This tells us that the problem does not occur while processing the current dialogue turn, but that it must stem from an error in processing a previous dialogue turn that had as a consequence that a second mentioned object was not recognised and therefore does not appear in the conversation memory. The user can then continue analysing the previous turns to retrieve the original source of the problem.

## Conclusion

This paper has introduced a novel methodology to solve visual dialogue tasks, based on the use of neuro-symbolic procedural semantic representations. Concretely, this methodology encompasses (i) the use of a conversation memory as a data structure that explicitly and incrementally represents the information that is expressed during the subsequent turns of a dialogue, and (ii) the representation of natural language expressions as neuro-symbolic semantic networks that are grounded in both visual input and the conversation memory. These networks are composed of a combination of subsymbolic primitive operations that model the perceptual capacities of an agent and symbolic primitive operations that model its reasoning capabilities. Upon evaluation on the MNIST Dialog and CLEVR-Dialog benchmarks, our methodology respectively yields state-of-the-art and near-state-of-the-art performance.

Our methodology presents four main advantages with respect to the state of the art in visual dialogue, which is dominated by attention-based neural network approaches. First of all, the methodology is to a great extent explainable in human-interpretable terms. The semantic networks that represent the meaning of natural language utterances are composed of human-interpretable primitive operations, their input and output arguments are either meaningful symbols or interpretable visual attentions, and the conversation memory represents information conveyed in earlier dialogue turns using a transparent symbolic data structure. This enables the human observer to verify whether an answer returned by the system is indeed the result of sound logic reasoning, as well as to trace back the exact source of any perception or reasoning errors that might occur. Second, the methodology avoids potential reasoning inconsistencies by implementing the primitive operations on top of a shared inventory of highly-specialised neural modules. This ensures at least that the results of different primitive operations are guaranteed to be consistent with each other, whether the neural modules have made correct predictions or not. Third, the system can effectively monitor its own performance, as errors that result from language processing or from the execution of individual primitive operations lead in many cases to an automatically detectable failure in the execution of a semantic network. Finally, the modularity of the approach ensures that new primitive operations can be dynamically added in order to accommodate new tasks or in order to model new cognitive capacities acquired by an agent. These new primitive operations can thereby build further on existing primitive operations or neural modules where appropriate.

Finally, the research reported on in this paper contributes to the growing body of research in artificial intelligence that tackles tasks that involve both low-level perception and high-level reasoning using a combination of neural and symbolic techniques. Neural techniques are used to deal with low-level perception tasks and thereby give rise to meaningful symbols that can then be used as a basis for higher-level reasoning operations. It thereby bears the promise of leading to the development of artificial agents with more explainable, consistent and human-like cognitive capacities.

## Supporting information

S1 Supporting information Training and evaluation details of neural modules (PDF)

## Footnotes

^1^ Somewhat counterintuitively, the answer to this question is a number and not a boolean value.

^2^ The evaluation of the model of the CLEVR dataset is reported by [71]

^3^ The evaluation of the model on the MNIST Dialog dataset is reported by [35] and of CLEVR-Dialog by [12]

^4^ This information was communicated to and acknowledged by the creators of the dataset.
