# Supplementary material for: Neuro-symbolic procedural semantics for explainable visual dialogue
Source: PLoS One. 2025 May 27;20(5):e0323098. doi: 10.1371/journal.pone.0323098 (PMC12112666; doi:10.1371/journal.pone.0323098)
Supplement: S1 Supporting information — (PDF) [file pone.0323098.s001.pdf]

## Supporting information

**S1 Appendix.** This appendix provides full details on the architecture and training regime of the neural modules underlying the subsymbolic primitive operations. The neural modules perform either instance segmentation (those used by the SEGMENT-SCENE operation) or binary classification (those used by the QUERY, FILTER, RELATE, EXTREME-RELATE and IMMEDIATE-RELATE operations).

**Modules performing instance segmentation.** The instance segmentation module is implemented through a Mask R-CNN model [1]. This module takes as input an image and returns a set of as many visual attentions as there are objects in the scene, with each attention highlighting one of the objects. The instance segmentation module is implemented using the Detectron2 framework [2]. It consists in a model for instance segmentation pretrained on the COCO images dataset [3], which we then finetuned on the CLEVR mini dataset [4]. For finetuning, we used a batch size of 8, a learning rate of 0.00025 and 10,000 iterations.

While the same architecture could also be used for processing the MNIST Dialog images, instance segmentation is not really an issue when it comes to this dataset. As all images consist of the same 4x4 grid layout, it is straightforwardly dividable into 16 visual attentions. As a consequence, we skip the instance segmentation module there.

**Modules performing binary classification.** The binary classification modules are trained to predict whether a specific conceptual categorisation holds for a given object or set of objects in a scene. The binary classifiers are each implemented by a convolutional neural network (CNN) adopting the SqueezeNet architecture [5] in its 1.1 version[1]. As the SqueezeNet architecture expects a single RGB image as input, our modules first combine their different inputs into a single tensor using a pre-encoding layer, as illustrated in Fig S1a for modules with two inputs (i.e. the colour, material, style, size, shape, number, bgcolour and extreme-relate modules) and in Figure S1c for modules with three inputs (i.e. the relate and immediate-relate modules). Both pre-encoding layers make use of a *DoubleConv* operation, which is shown in Figure S1b. A DoubleConv operation consists in two *Conv* operations, which each consist in a convolutional layer with kernel size 3 and padding 1, followed by a batch normalisation operation [6] and a rectified linear unit (ReLU). In general, the first Conv operation in this DoubleConv operation changes the number of channels while the second Conv operation keeps the number of channels the same.

When pre-encoding two inputs, each of the inputs first passes through a DoubleConv layer. If the input is an attention, the first Conv in this DoubleConv layer increases the number of channels from 1 tot 3. If the input is an image, the number of channels does not change. Then, the resulting feature maps are combined using element-wise multiplication, which results in a $3xWxH$ tensor. When pre-encoding three inputs, the image and the two attentions each pass through a distinct DoubleConv layer, which increases the number of channels from 1 to 3 for the attentions and keeps the number of channels the same for the image. Each of these DoubleConv operations is followed by a max pooling operation, which halves the width and height dimensions of the feature maps. Then, the resulting feature maps of the image are concatenated with the resulting feature maps of the first attention. The concatenation then passes through another DoubleConv layer, which doubles the number of channels from 6 to 12. The same steps apply for the image and the second attention. The two resulting tensors are then concatenated and passed a final time through a DoubleConv layer, which reduces the number of channels to three, resulting in a $3xW_{/2}xH_{/2}$ tensor.

The result of the pre-encoding layer is then passed to the SqueezeNet architecture, followed by a LogsSoftMax layer. The output is binary and consists of predictions for the labels 'yes' and 'no'. The overall architecture of the modules is shown in Fig S2.

In order to train and evaluate the neural modules, we have generated one dataset per module per benchmark. For CLEVR-Dialog, we have used images 0-59,999 of the original training set as training data and images 60,000-69,999 of the original training set as validation data. Then, we have used

[1] `https://github.com/forresti/SqueezeNet/tree/master/SqueezeNet_v1.1`

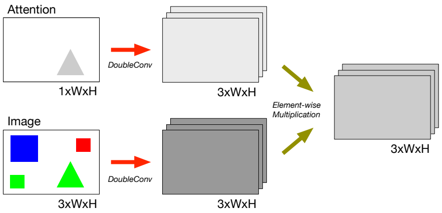


(a) The architecture of the pre-encoding layer with two inputs.

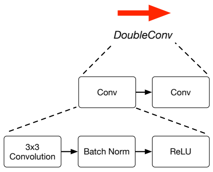


(b) A DoubleConv layer consists in two Conv layers, each consisting in a 3x3 convolution, followed by a batch norm and a ReLU.

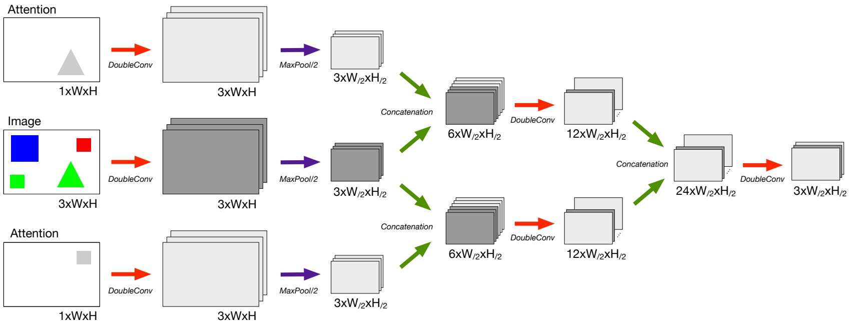


(c) The architecture of the pre-encoding layer with three inputs.

Figure S1: **Schematic overview of the pre-encoding operations.**

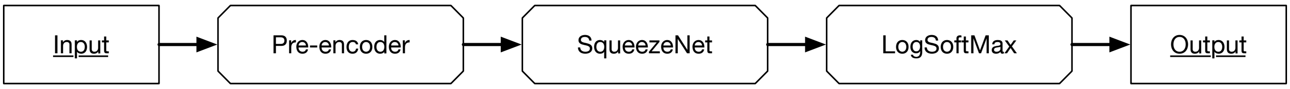


Figure S2: **The architecture of the modules.** The input goes through a pre-encoder, then through the SqueezeNet architecture, followed by a LogSoftMax layer.

the instance segmentation module described above to find all instances of objects in these images. We have then computed the Euclidean distance between the coordinates specified in the meta-data and the coordinates of the predicted instances in order to link the object instances to their symbolic description in the meta-data of the dataset. The result is a dataset in which each predicted instance (in the form of a visual attention) is accompanied by a symbolic description of its attributes. Based on this dataset, one dataset per module was then generated, which consists of instances annotated with a label 'yes' or 'no'. The correct label is found via the symbolic meta-data. A total of 28 datasets were generated, one for each CLEVR-Dialog module.

For MNIST Dialog, the dataset generation process was similar. We used the 30,000 images from the training set as training data and the 10,000 images from the validation set as validation data. First, we found all instances in the images by dividing the image into 16 attentions. Then, we linked these instances to their symbolic description using the index provided in the meta-data. This resulted in a dataset with all instances paired with their symbolic description. Based on this dataset, we generated one dataset per module, with consists in all the instances accompanied by a label that indicates whether the instance has the attribute or not. A total of 26 datasets were generated, one for each of the MNIST Dialog modules.

The hyperparameters used for training the CLEVR-Dialog modules are the following: batch size 128, learning rate 0.0001, and negative log likelihood (NLL) as the loss function. For the MNIST Dialog modules, we used a batch size of 256, a learning rate of 0.0001, and negative log-likelihood (NLL) as the loss function. In total, we trained 28 modules for CLEVR-Dialog and 26 modules for MNIST Dialog. An overview of the loss and the accuracy on the validation set is given in Table S1.

For training the modules, we used the HPC infrastructure provided by the Vlaams Supercomputer

Table S1: **Overview of the loss and accuracy of the CLEVR-Dialog and MNIST Dialog modules.**

| **CLEVR Modules** | **Loss** | **Accuracy** | **MNIST Modules** | **Loss** | **Accuracy** |
|---|---|---|---|---|---|
| colour-blue? | 0.0011 | 99.99 | colour-blue? | 0.0020 | 99.97 |
| colour-brown? | 0.0033 | 99.95 | colour-red? | 0.0072 | 99.93 |
| colour-cyan? | 0.0019 | 99.98 | colour-green? | 0.00 | 100.0 |
| colour-gray? | 0.0039 | 99.97 | colour-violet? | 0.0013 | 99.98 |
| colour-green? | 0.0027 | 99.97 | colour-brown? | 0.0060 | 99.81 |
| colour-purple? | 0.0011 | 99.98 | style-stroke? | 0.00 | 100.00 |
| colour-red? | 0.0038 | 99.96 | style-flat? | 0.00 | 100.00 |
| colour-yellow? | 0.0061 | 99.95 | bgcolour-white? | 0.00 | 100.00 |
| shape-cube? | 0.0089 | 99.88 | bgcolour-cyan? | 0.00 | 100.00 |
| shape-cylinder? | 0.0093 | 99.80 | bgcolour-salmon? | 0.00 | 100.00 |
| shape-sphere? | 0.0072 | 99.90 | bgcolour-yellow? | 0.00 | 100.00 |
| size-small? | 0.0079 | 99.92 | bgcolour-silver? | 0.00 | 100.00 |
| size-large? | 0.0101 | 99.91 | number-0? | 0.0130 | 99.95 |
| material-metal? | 0.0089 | 99.90 | number-1? | 0.0049 | 99.85 |
| material-rubber? | 0.0084 | 99.91 | number-2? | 0.0040 | 99.95 |
| relate-behind? | 0.0050 | 99.91 | number-3? | 0.0009 | 99.98 |
| relate-left? | 0.0014 | 99.97 | number-4? | 0.0080 | 99.90 |
| relate-right? | 0.0021 | 99.97 | number-5? | 0.0101 | 99.66 |
| relate-front? | 0.0034 | 99.92 | number-6? | 0.0019 | 99.92 |
| immediate-relate-behind? | 0.0091 | 99.82 | number-7? | 0.0075 | 99.88 |
| immediate-relate-left? | 0.0037 | 99.89 | number-8? | 0.0016 | 99.97 |
| immediate-relate-right? | 0.0034 | 99.91 | number-9? | 0.0037 | 99.94 |
| immediate-relate-front? | 0.0068 | 99.84 | immediate-relate-above? | 0.00 | 100.0 |
| extreme-relate-behind? | 0.0114 | 99.78 | immediate-relate-below? | 0.00 | 100.0 |
| extreme-relate-left? | 0.0029 | 99.93 | immediate-relate-left? | 0.00 | 100.0 |
| extreme-relate-right? | 0.0047 | 99.95 | immediate-relate-right? | 0.00 | 100.0 |
| extreme-relate-front? | 0.0009 | 99.98 | | | |
| extreme-relate-middle? | 0.0835 | 97.59 | | | |

Center (VSC) with modern CPU (Intel Xeon) and GPU (Nvidia Tesla P100, Nvidia A100, Nvidia Volta V100) platforms.

## References


[1] He K, Gkioxari G, Dollar P, Girshick R. Mask R-CNN. In: Cucchiara R, Matsushita Y, Sebe N, Soatto S, editors. 2017 IEEE International Conference on Computer Vision (ICCV). Washington, D.C., USA: IEEE Computer Society; 2017. p. 2961–2969.

[2] Wu Y, Kirillov A, Massa F, Lo WY, Girshick R. Detectron2; 2019. `https://github.com/facebookresearch/detectron2`.

[3] Lin TY, Maire M, Belongie S, Hays J, Perona P, Ramanan D, et al. Microsoft COCO: Common objects in context. In: Fleet D, Pajdla T, Schiele B, Tuytelaars T, editors. European Conference on Computer Vision. Cham, Switzerland: Springer; 2014. p. 740–755.

[4] Yi K, Wu J, Gan C, Torralba A, Kohli P, Tenenbaum J. Neural-symbolic VQA: Disentangling reasoning from vision and language understanding. In: Bengio S, Wallach H, Larochelle H, Grauman K, Cesa-Bianchi N, Garnett R, editors. Advances in Neural Information Processing Systems 31 (NeurIPS 2018). Red Hook, NY, USA: Curran Associates Inc.; 2018. p. 1031–1042.

[5] Iandola FN, Han S, Moskewicz MW, Ashraf K, Dally WJ, Keutzer K. SqueezeNet: AlexNet-level accuracy with 50x fewer parameters and <0.5MB model size. arXiv preprint arXiv:160207360. 2016;.

[6] Ioffe S, Szegedy C. Batch normalization: Accelerating deep network training by reducing internal covariate shift. In: Bach F, Blei D, editors. Proceedings of the 32nd International Conference on Machine Learning. Cambridge, MA, USA: PMLR; 2015. p. 448–456.
